# Perceptions of Football Analysts Goal-Scoring Opportunity Predictions: A Qualitative Case Study

**DOI:** 10.3389/fpsyg.2021.735167

**Published:** 2021-09-06

**Authors:** Rubén D. Aguado-Méndez, José Antonio González-Jurado, Álvaro Reina-Gómez, Fernando Manuel Otero-Saborido

**Affiliations:** ^1^Departamento de Deporte e Informática, Facultad de Ciencias del Deporte, Universidad Pablo de Olavide, Seville, Spain; ^2^Watford Football Club, Watford, United Kingdom

**Keywords:** match analysis, coaches, qualitative research, style of play, tactics

## Abstract

This study aimed to understand the way tactical football analysts perceive the general match analysis issues and to analyze their tactical interpretation of the predictive models of conceded goal-scoring opportunities. Nine tactical analysts responded to the semi-structured interviews that included a general section on the match analysis and a specific one on the results of a study on goal-scoring opportunities conceded by a Spanish La Liga team. Following their transcription, the interviews were codified into categories by the two researchers using Atlas Ti® software. Subsequently, frequency count and co-occurrence analysis were performed based on the encodings. The content analysis reflected that analysts play a crucial role in the analysis of their own team and that of the opponent, the essential skills to exercise as a tactical analyst being “understanding of the game” and “clear observation methodology.” Based on the case study of the conceded goal-scoring opportunities, the major causes and/or solutions attributed by analysts in some of the predictive models were the adaptability of the “style of play” itself according to the “opponent” and “pressure after losing.”

## Introduction

In recent years, there are great technological advances in the analysis of football performance (Sarmento et al., 2017). Despite this progress, still there is a gap between the scientific community and the knowledge that the technical bodies of professional clubs actually need to acquire (Carling et al., 2013). A reason for this gap could be the low amount of research that combines both quantitative and qualitative analyses (Sarmento et al., 2020). Though video analysis plays a key role for coaches to improve the performance of their own team and to analyze the opponents (Wright et al., 2012), professional football coaches still encounter problems that have yet to be addressed by research (Wright et al., 2014).

There is one suitable tool that brings data and scientific research closer to answering the tactical questions set out by the technical bodies: the “mixed methods” technique (Sarmento et al., 2013a). This methodology is defined by Johnson and Onwuegbuzie (2007) as a way of conducting research that combines quantitative and qualitative research elements. According to Onwuegbuzie (2012), it can provide added value by giving a more holistic overview of the collected data and by contextualizing the conclusions drawn from the quantitative analysis (Harper and McCunn, 2017). Therefore, the quantitative-qualitative combination is a useful way of identifying not only “what happens in a match,” but also “why it happens,” based on the interpretations made by the professionals of the sport in question (Halperin, 2018). Based on all the above, and because of the holistic nature that data triangulation brings to a study, *mixed methods* can be described as contributing more than the mere sum of qualitative plus quantitative approaches (Fetters and Freshwater, 2015).

The qualitative research method, such as interviews with the coaches and analysts, could help to develop practical applications of research in game analysis. Despite the extensive literature existing on the match analysis (Hughes, 2008; Carling et al., 2009), few studies have collected the opinions of coaches. The evidence that has been collected, for example, on the game observations of the coaches (Sarmento et al., 2013b), the role of performance analysis (Mackenzie and Cushion, 2013), and their opinions regarding detected game patterns (Sarmento et al., 2016) is still insufficient compared to a large number of quantitative football studies.

Qualitative case studies make it easier for the coaches to actually apply scientific findings. Indeed, the results are related to the real and specific technical-tactical contexts rather than being generalized (Ruddock et al., 2019). In this sense, the present qualitative study used as a reference, the results of a study on the Spanish La Liga team characterized by a high ball-possession profile. The study concluded by advancing a prediction of the conceded goal-scoring opportunities, based on the contextual, defensive, and offensive variables (Aguado-Méndez et al., 2020b). Therefore, the objectives of the present study were as followed:

- First, to understand how the specialists participating in the study perceived the game analysis with respect to their own team and the opponents.
- And second, to examine the tactical interpretation of the analysts of the quantitative data based on a study that focused on conceded goal-scoring opportunities.

## Materials and Methods

### Design

The selected methodology was qualitative using the semi-structured interviews with open-ended responses (Smith and Caddick, 2012).

### Participants

The participants were nine football analysts. Of the nine participants, seven were working for the professional teams (Liga Santander, Liga Smartbank), one was part of the Second Division B team of Spain (Segunda División B), and the other was part of the Third Division team (Tercera División) during the 2019–2020 season. They were all in possession of the senior title of football coach and had served as such throughout their career. The study protocol was approved and followed the guidelines stated by the Ethics Committee of the Research Center of Sport Sciences at University Pablo de Olavide, based at Seville (Spain) and conformed to the recommendations of the Declaration of Helsinki.

### Instruments

A semi-structured interview comprising of a general section and a specific section was designed by three game analysis research experts (Bardin, 2008). Questions in the general section (Table 1) were based on the work of Sarmento et al. (2015). This part of the interview includes general game analysis questions that can be used both for the own team analysis and the opponents.

**Table 1:** Questions and categories in the general section of the interview.

**Category: General game analysis**
**Questions**	**Categories**
1. Importance of analyzing matches	Importance of own team analysis
	Fundamental
2. Analyst features	Ability to synthesize
	Understanding of the game
	Clear observation methodology
	Objectivity
3. Most important aspects to analyse matches	Adapting to the coach
	Phases of the opponent game
	Opponent's strong and weak points
4. Data provider information	Relevance	Distances
		Contextual factors: minute
		Goal-scoring opportunities
	Problems	Provider heterogeneity
		Overly long reports
	Future	Custom data
		Prediction/probability

Questions in the specific section asked the analysts about the results of the study of Aguado-Méndez et al. (2020a). This latter work produced four predictive models of conceded goal-scoring opportunities based on the contextual, defensive, and offensive variables in a 2017/2018 Spanish league case study.

The results of four models were:

- Model 1: the probability of conceding goal-scoring opportunities after “own half losing” was four times greater if this took place between 0′-15′ and 46′-60′ than between 76′ and 90′.
- Model 2: the probability of conceding a goal-scoring opportunity after “own half losing” was almost three times greater if the opponent only gave 0 or 1 pass to finish the move than if it gave five or more passes take place.
- Model 3: the probability of conceding a goal-scoring opportunity in the second half was three times greater if the loss of the ball took place by a “steal” than by a “forced mistake.”
- Model 4: the probability of a goal-scoring opportunity conceded being a goal was almost half as likely to result in a draw as in a win.

In the present research, the analysts were asked about the “causes” and “solutions” they would offer as analysts in each of the four models (Table 2).

**Table 2:** Questions and categories of the specific section of the interview based on the four predictive models of conceded goal-scoring opportunities.

**Model 1: Minute and loss zone**	
**Questions**	**Categories**
**Cause**	**Concentration**
	**Minute—Loyalty to coach ideas**
	**Style of play**
	**Opponent**
**Solution**	**Concentration**
	**Style of play**
	**Pressure after losing**
**Model 2: Duration and loss zone**	
**Questions**	**Categories**
**Cause**	**Physical aspects**
	**Opponent players participating/counterattack speed**
	**Team positioning**
	**Pressure after loss**
**Solution**	**Style of play**
	**Pressure after losing**
**Model 3: Steal and minute**	
**Questions**	**Categories**
**Cause**	**Physical aspects**
	**Concentration**
	**Style of play**
	**Opponent**
	**Concentration**
	**Pressure after losing**
**Solution**	**Style of play**
	**Pressure after losing**
**Model 4: Match status**	
**Questions**	**Categories**
**Cause**	**Concentration**
	**Style of play**
	**Opponent**
**Solution**	**Style of play**
	**Opponent**
	**Scoreboard conditioned tasks**

### Category System

**Figure 1:**
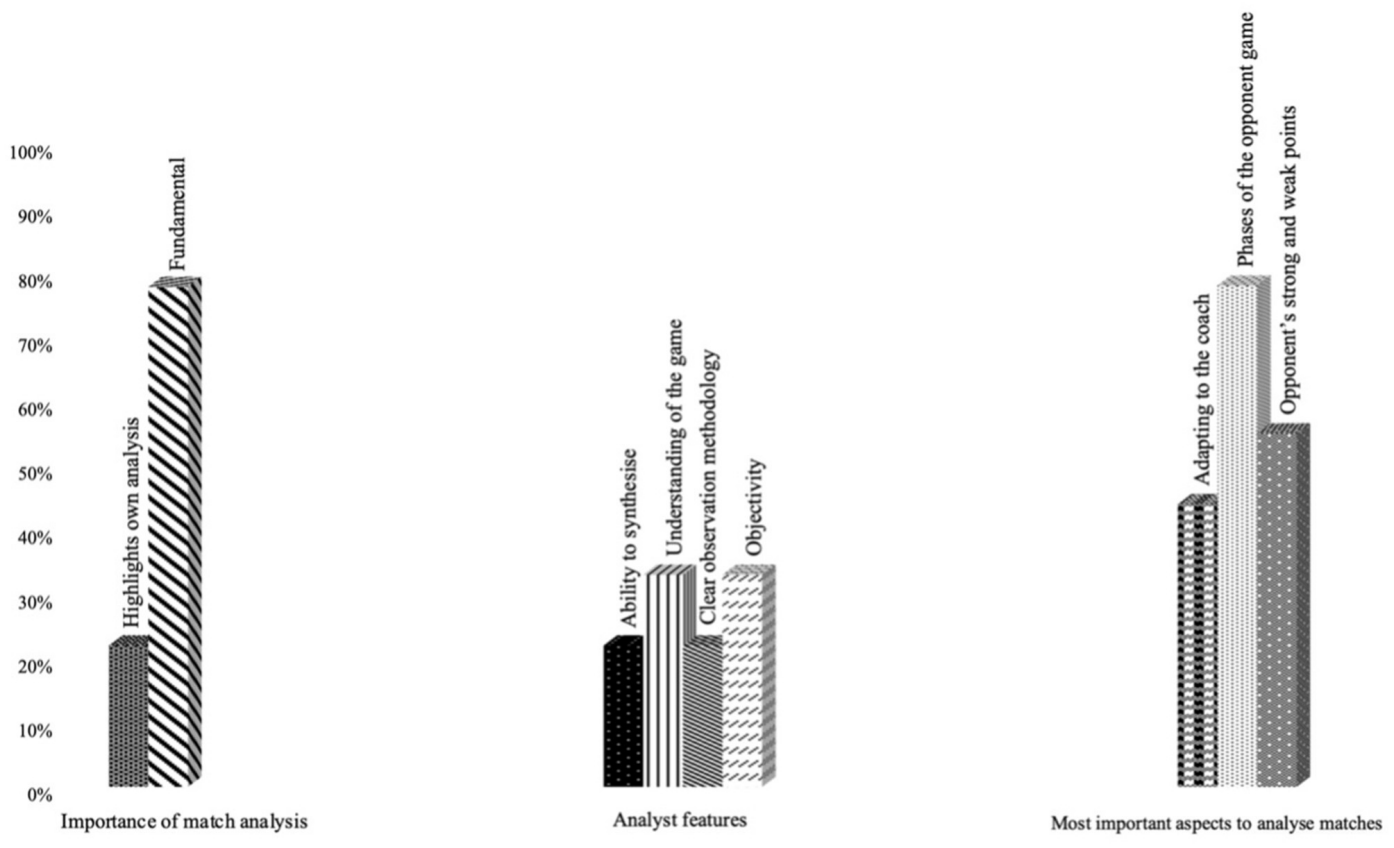
Answer frequency of the categories of the first three questions for Objective 1.

A category system was first elaborated (González et al., 2020) following the steps established by Braun and Clarke (2006). To test it, three interviews were coded based on the predefined category system. Following this pilot coding, the system was configured based on five distinct parts (one for the general section with four questions, and four others with questions on the cause and solution of each model of the study that they were being asked about). After analyzing the interviews, the most frequent answers were established as categories. Figure 1 shows the relationship between the questions of the specific section and its categories.

### Data Collection

Interviews were conducted and recorded *via* videoconference with experts. Coaches previously received a video summary of the analysis data they were going to be asked about. Each interview lasted for approximately 45 min and recordings were made for the subsequent transcription and analysis in Atlas TI version 8.4.5. The analysts were given time to clarify their thoughts and rewrite their answers.

The authors confirm that the data supporting the findings of this study are available within the article and/or its Supplementary Material. This study has followed the Consolidated criteria for reporting qualitative research (COREQ) checklist.

### Materials

The interviews were recorded *via* videoconference using the official Blackboard Collaborate Ultra platform of the University Pablo de Olavide, Seville, Spain after obtaining the consent from the participants. The NCH Express Scribe® professional software was used to transcribe the recorded interviews. Finally, Atlas.ti 8.4.5® software was used for the content analysis of the data obtained in the study.

### Procedure and Analysis

First, the data were briefly analyzed by transcribing, reading, and noting the initial ideas, establishing a code map per question and category. The codes were then classified under possible themes. Third, a thematic map was constructed based on the coded data to visually analyze the themes and the relationships between them. Once all the content of the interviews was encoded, a frequency count, and a co-occurrence analysis technique of the categories were applied in order to determine the associations between the different categories and/or questions.

## Results

The first objective was to know the general perceptions of the analysts toward the game analysis of their own team and that of the opponent. To do this, categories were analyzed using the Atlas.ti version 8.4.5 software using the co-occurrences table based on the questions in Table 1.

Regarding the first question “what importance do you give to the analysis?,” a total of seven of the nine analysts agreed to describe the analysis as a “Fundamental” process (Figure 1).

*The analysis is our point of departure to know what we have and where we are. From this point onwards, we can plan, trainm and re-analyse whether the objectives have been achieved. It is thus essential (participant 4)*.

Likewise, when asked about the essential characteristics of an analyst, the answer frequency (Figure 1) underscored “Clear observation methodology” and “Understanding of the game” as the most important attributions.

*A good observational methodology is essential for analysts, it implies appropriate control over the data's quality, avoiding observation biases, understanding what is truly observable, validity, precision, objectivity and reliability (participant 4)*.

The aspects that were considered to be most important when analyzing the opponent teams, the participants highlighted the fact of knowing “Phases of the opponent's game” and their “Strengths and weaknesses,” and all this “Adapted to the coach's ideas.”

*The first thing is to know the game and one's own team, as well as what the coach wants from his team, to be able to show the game patterns and the opponent's strengths/weaknesses compared to what one's own team can offer (participant 5)*.

**Figure 2:**
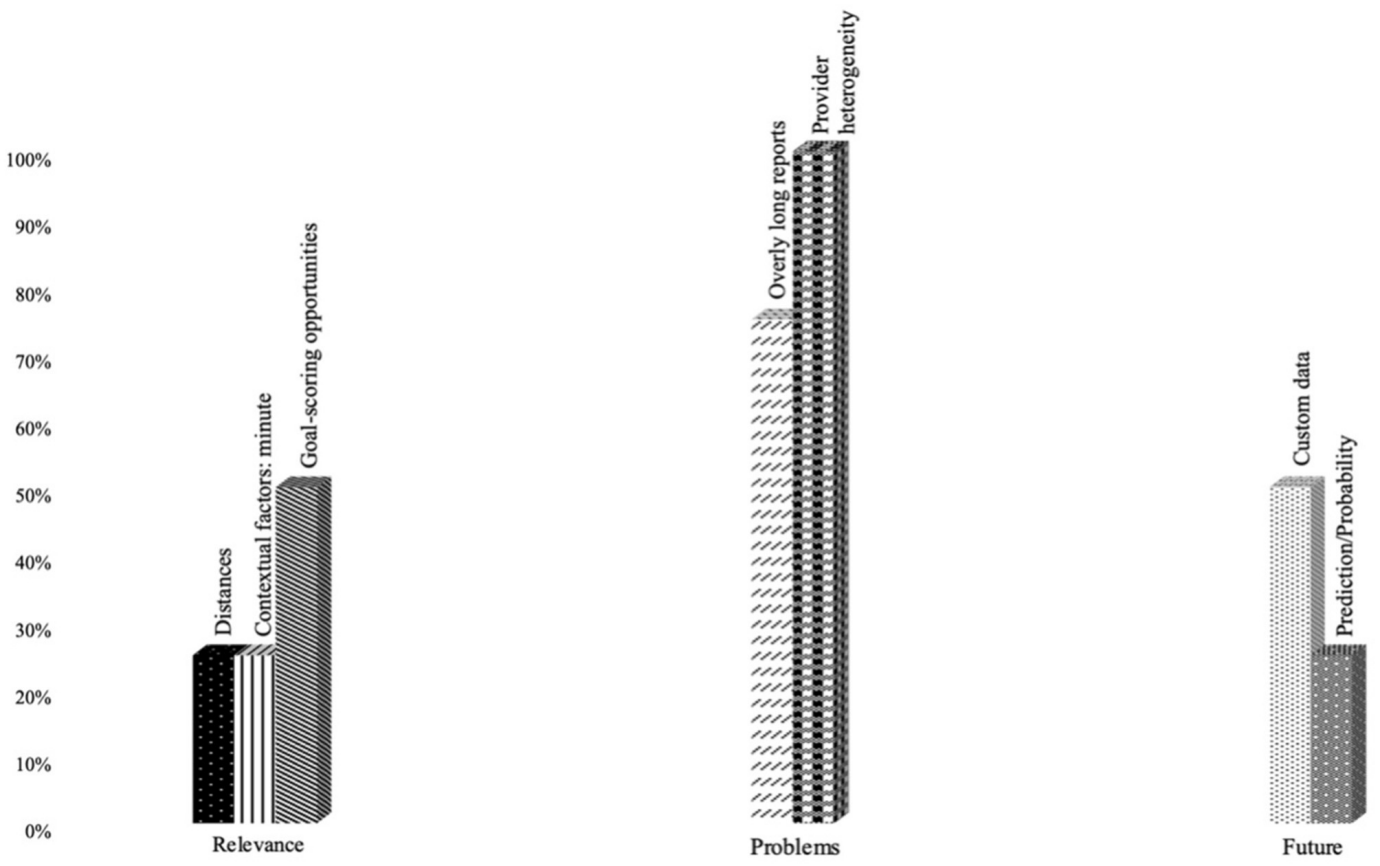
Answer frequency to the question “Data provider information.”

With regard to the assessment of data provider information by the analysts, participants answered three questions: “relevance”, “problems,” and “future” (Figure 2).

In the case under study, analysts considered “Goal-scoring opportunities” to be the most relevant performance indicator among those offered by the data providers.

*The most important thing is the way, quantity and quality of the goal-scoring opportunities generated by the team and what happens after. That is what I attach the most importance to when the match ends, because when you start to improve that aspect, you get closer to achieving results (participant 2)*.

Regarding the problems, the analysts agreed on the prominence of two issues: “Overly long reports” and “Data heterogeneity.”

*I am very critical of such long reports because despite the great quantity of data, it does not mean that it provides the information you need (participant 4)*.

Regarding “Heterogeneity among suppliers” the response of interviewee six was highly illustrative:

*We should define the concept of goal opportunity more clearly for data providers. We encountered the problem that for a provider, a move was a clear goal opportunity: a high xG was attributed but not so much in the case of another, the value being lower. Therefore, greater uniformity is needed both for opportunities and in all performance indicators (participant 6)*.

Answers on the prospective “future” were organized in two dimensions. First, “custom data” adapting it to the characteristics of each team, second, “predict the opponent's behaviors according to the phase of the game.”

*A great breakthrough would be the adding of probabilities, to predict what can happen in certain situations. I think that means and percentages describe what has already happened and cannot be changed, but ideally, it should anticipate what can happen in certain situations so we can work on it (participant 6)*.

The second objective of this study was to “examine the analysts' tactical interpretation of a study's quantitative data on conceded goal-scoring opportunities.” The questions posed to the analysts concerned the four predictive models about the conceded goal-scoring opportunities. Participants were asked about the “causes” and “solutions” in the case of each model (Table 2).

**Figure 3:**
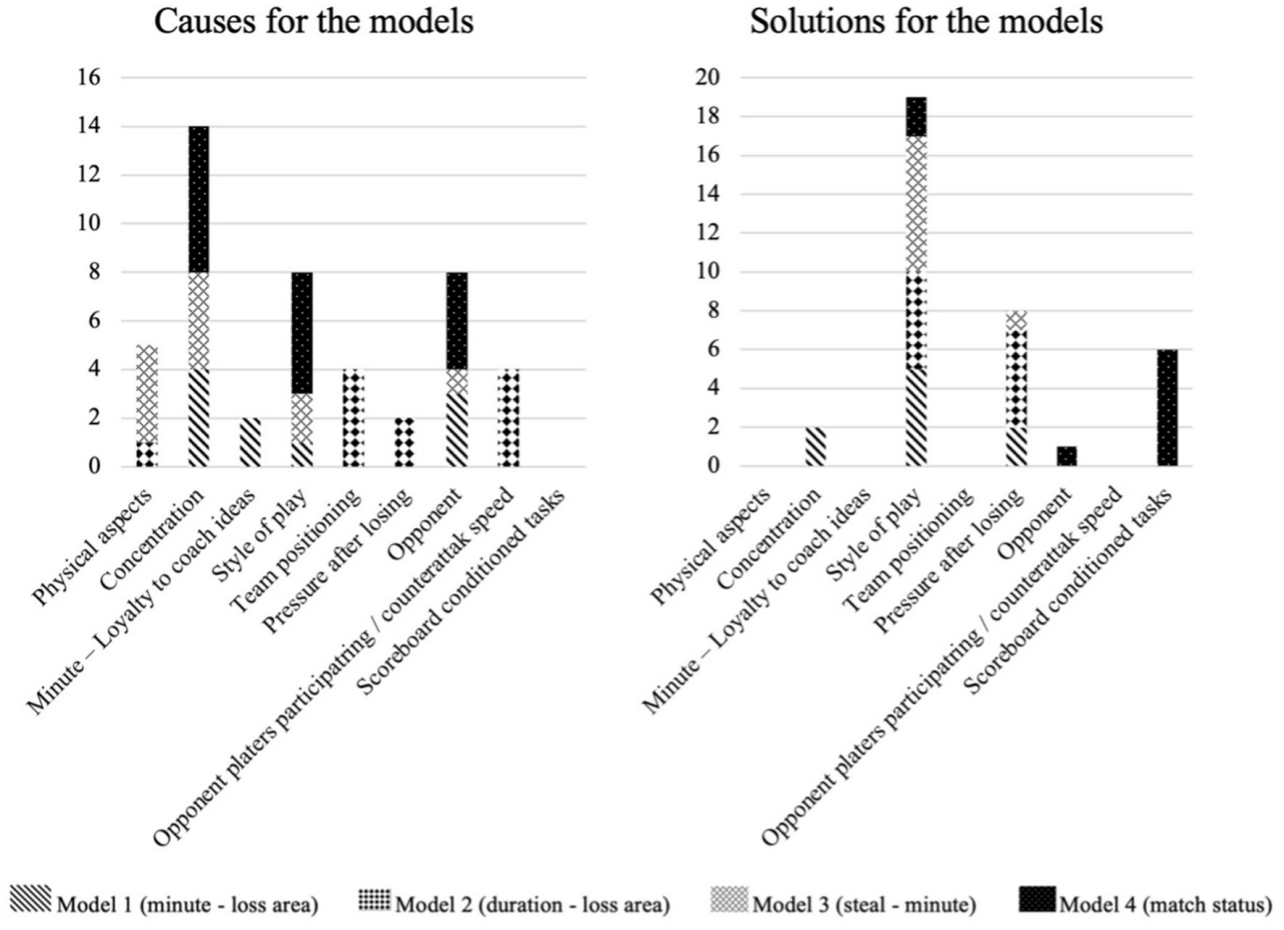
Frequency of causes and solutions in all four predictive models.

A first descriptive analysis (Figure 3) shows that the causes most frequently given in the four predictive models were “Concentration” (14) “Style of play,” (8) and “Opponent” (8). Other causes that obtained a lower percentage were “Conditioning factors,” “Team positioning,” and “Opponents taking part/counterattacking speed.” With regard to the “solutions” proposed by analysts in each predictive model, “Style of play” (19), “Pressure after losing” (8), and “Exercise with conditioner score” (6) obtained the highest response rates. The variables “Concentration” (2) and “Opponent” (1) were the lowest mentioned solutions.

**Figure 4:**
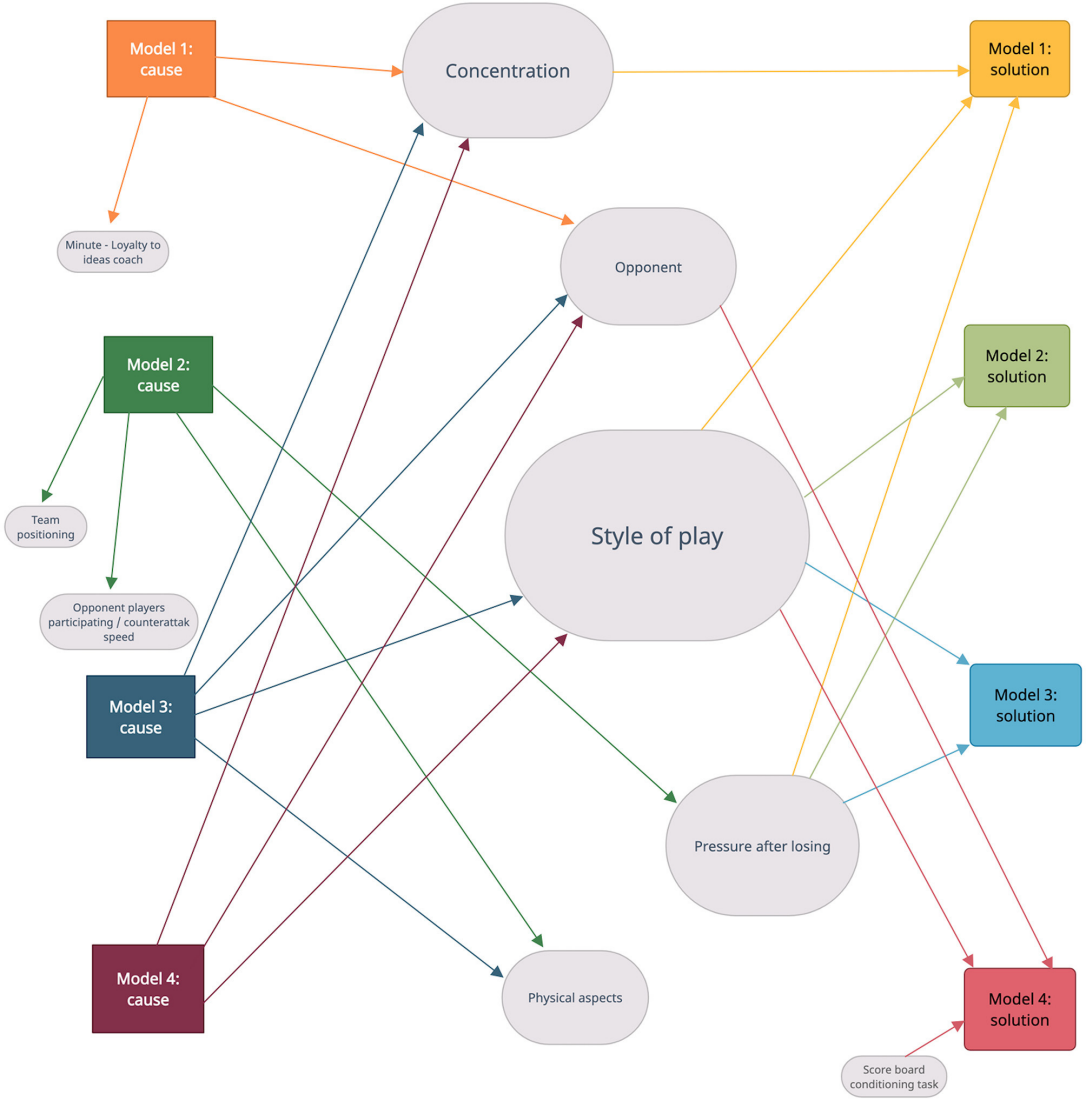
Relationship between causes and solutions in the four predictive models.

Moreover, beyond a quantitative analysis, the co-occurrence analysis technique allowed the design of a network of relationships between “causes” and “solutions” in the four predictive models (Figure 4).

The “Style of play” category seems to be the most decisive for analysts as it is included in both the “causes” (in model 1, 3, and 4) and “solutions” (for all four models). A highly representative example is the response of interviewee four when asked about the solution in model 1. This model predicted four times greater chance of receiving a goal-scoring opportunity after losing in his own half at intervals 0′-15′ and 45′-60′ than in 75′-90′) (Aguado-Méndez et al., 2020a).

*One form of intervention may be to change the system. In addition*,*I would do conditional tasks to provoke movements and situations to my advantage. We also modify these rules according to the exercise's outcome (participant 4)*.

Likewise, according to responses of analysts, the “opponent” category was considered an important cause. The interviewees placed this category as a “cause” for three models (1, 3, and 4), as well as the “solution” in model 4. In this model, it was predicted that it was two times more likely that the conceded opportunity would end in a goal with a favorable result rather than a tie. An example of how the “opponent” influences this model is exemplified by participants four and six.

*Real Betis team, even when leading on the scoreboard, did not changes its idea of play very much, while the opponent was taking steps forward to achieve a tie, and that can lead to receiving more goal-scoring opportunities (participant 6)*.

On the other hand, the “pressure after losing” category was the “cause” in model 2, but above all, it stands out as a “solution,” since it was proposed for this purpose in models 1, 2, and 3. An example of this solution was proposed by analysts two and nine in model 2. This model predicted a higher chance of receiving a goal-scoring opportunity when the ball was lost in own half and, at the same time, few players were involved in the move; therefore, its duration was reduced.

The team should work to achieve the necessary resources and get organized around the ball to rally as much as possible in the face of possible losses (participant 2).

In addition, another major category under which the psychological or emotional aspects could be organized was “concentration.” It was cited by the analysts as “cause” for three models (1, 3, and 4) and as a “solution” in two models (1 and 3). The interviewees eight and three interpreted that concentration was a “cause” in model 3 of Aguado-Méndez et al. (2020a). This model predicted that the probability of receiving a goal-scoring opportunity by stealing a ball was two-times as high than by a bad pass due to pressure in the second half.

*It may be due to over-relaxation during those minutes. Perhaps the fact of adopting a very horizontal style, pausing a lot when in possession of the ball also led to a lack of energy during the match (participant 8)*.

Finally, the “physical aspects” category was the “cause” of the opportunities conceded in two models (2 and 3). An example of why this factor was the origin in model 3 (it related form of loss and minute) was given by analyst four.

*As fatigue sets in, players make worse decisions, and a typically bad decision they usually make is that the more tired they are, the more they keep the ball, the more they play 1 against one, and the lesser support they get from their teammates—because they are also tired and that is why it causes those losses. (participant 4)*.

## Discussion

The objectives of this study were two-fold: to gather the overall perceptions of the interviewed analysts on the game analysis of their own team and the opponent team, and to analyze the tactical interpretation of the results of a study on goal-scoring opportunities. Regarding the first objective, analysts answered general questions about the analysis of the game of their own team and the opponent team. The content analysis of the responses showed that they believed that the analysis of both their own team and the opponent was important. A total of seven of the nine participants all rated the analysis of the game as “fundamental.” Supporting this “fundamental” nature of the analysis, in recent years, scientific publications have been highlighting match analysis as a way of optimizing the preparation phase of competitions (Hughes and Franks, 2004) and of building an understanding of the complexity of sports (Brito Souza et al., 2019).

The “Understanding of the game” and a “Clear observation methodology” were considered as essential skills for the tactical analysts. This result is consistent with various works that have underscored the key role of disposing of a good observation tool to collect information in a structured way using the predefined categories (Sarmento et al., 2013b). The “Phases of the game” and the “Strengths and weaknesses” were regarded as the major aspects to study when analyzing the opponent team. These results support the study of Carling et al. (2008). In addition, it has been shown that once the coach obtains this data, using this information in mobile sessions helps players to better know and understand their opponent (Carling et al., 2005).

Moreover, within this first objective of understanding the perceptions of analysts, they considered “Goal-scoring opportunities” as the most relevant indicator of match analysis performance. This opinion is in line with the specialized literature that considers that, due to their greater frequency, goal-scoring opportunities are a better indication of football performance than the number of goals (Reina and Hernández-Mendo, 2012; González-Ródenas et al., 2016; Pratas et al., 2018). Analysts have, however, unanimously considered that no performance indicator is actually useful for coaches if it is included in “overly long reports.” In relation to this problem, Castelo (2009) points to the importance that analysts select the most relevant information, adopting a global perspective, so that it can be useful to prepare for the matches.

Regarding the future of game analysis, participants considered it to be the key to “customize the data” in the future, by adapting it to the characteristics of their own team as well as “predicting opponent behaviors” according to the phase of the game. A number of studies have already been carried out in which different variables have been studied to predict the probability of scoring a goal (Tenga et al., 2010), of being conceded a goal-scoring opportunity (Aguado-Méndez et al., 2020a), and the result of the match (Lago-Peñas et al., 2011).

Regarding the second objective, the participants interpreted the predictive models obtained in the study of Aguado-Méndez et al. (2020a). These models were obtained after analyzing the conceded goal-scoring opportunities of the Real Betis in the Spanish league 2017/2018 according to contextual, defensive, and offensive variables through a validated instrument of observational methodology.

The quantitative data of the predictions found in the mentioned study answer the questions: “what is happening” and “what could happen” in the match. On the other hand, understanding the functional logic of the game plays a major role in match analysis. Therefore, the interpretation made by the interviewees of the causes of those predictive models could help to answer the question of “why they happen” within the style of play of a particular football team.

The network of relationships between “causes” and “solutions” indicated that the “Style of play” was the most decisive category across all the models (Figure 1). Hewitt et al. (2016) define the style of play as a characteristic pattern of a team cutting across all the five moments of the game (offense, offensive transition, defense, defensive transition, and set pieces). Describing and measuring the different styles of play that soccer teams can adopt during a match is a very important step toward a more predictive and prescriptive performance analysis (Lago-Peñas et al., 2018). According to the patterns shown in these five moments of the game, teams can be defined, and associated with the performance indicators (Fernández-Navarro et al., 2016; Gómez-Ruano et al., 2018). For analysts, the “cause” in models 1, 3, and 4 (Table 2) was not adapting the “style of play” of this team—that had an associative style—to the contextual variables of the match. The interaction between the contextual variables as the match status and quality of opposition, and venue and quality of opposition were studied by Fernández-Navarro et al. (2018) determining its influence in styles of play in soccer match play. Further, in a study that analyzed the style of play of the 20 teams of La Liga in Spain in the 2016–2017 season, Castellano and Pic (2019) concluded that the realities of the competition forced the teams to adapt to contextual variables (opponent, location, position in the classification, etc.) in order to succeed. Consequently, it seems advisable to direct the training toward developing the flexible and adaptable styles of play to intra- and inter-match dynamics. It is thus unsurprising that the participants determined the modification of the “style of play” as a “solution” in the four predictive models, adapting to the opponent and the evolution of the outcome of the match to achieve successful results.

Directly related to the above, the analysts considered the “opponent” category as a major “cause,” as it was the originator in the three models (1, 3, and 4). This latter finding is in accordance with the study of Lago-Peñas (2009) that found the opponent team does change the way they play the match according to the minute, result, place, etc., thus succeeding in creating difficulties for the team that does not manage to adapt in the same way. The “Pressure after loss” category, i.e., the defensive transition, stood out as a “solution” in models 1, 2, and 3. The importance of properly performing the defensive transitions was reported in a study conducted on the German Bundesliga. This latter work showed that the best teams regained possession faster than lower-level teams (Vogelbein et al., 2014). In addition, the “concentration” category, which encompasses the psychological or emotional aspects, was a “cause” in three models (models 1, 3, and 4) and a “solution” in models 1 and 3. In this line, the specialized literature has previously shown how psychological factors can affect the performance of football teams (Pain and Harwood, 2007).

Finally, the “Physical aspects” category was the “cause” of the goal-scoring opportunities conceded in models 2 and 3. Indeed, this category affects the ball action accuracy as well as decision-making, because it reduces the precision of ball actions and dexterity generally. The review by Alghannam (2012) shows how physical performance decreases throughout the match due to accumulated fatigue. However, a recent study shows that if we consider effective time (i.e., not counting interruptions) rather than total playing time, the differences in terms of distances traveled at different speeds are much smaller between the first and second half of the match (Rey et al., 2020). In addition, the distance traveled at a high intensity (21–24 km/h) and sprint (>24 km/h) between the first and second part of the match depends on the demarcation, with midfielders and attackers decreasing their performance in the second half. Therefore, fatigue could explain the increase in ball inaccuracy as well as the worse decision-making of the players. But it would be incorrect to conclude that a cause-and-effect relationship exists between these factors. In any event, the contributions of qualitative studies based on the opinions of football professionals are essential to build an understanding of the quantitative data, not only regarding the “physical aspects” category, but also the functional logic of the game, globally (Wright et al., 2014, 2016; Sarmento et al., 2015, 2020).

## Conclusions

The objectives of the present study were first, to explore the general perceptions of the analysis of own team as well as of the opponent team, and second, to analyze the tactical interpretation of the quantitative data based on a study on conceded goal-scoring opportunities. The analysts who participated in this study found that it was fundamental to analyze their own team and the opponent team. Indeed, the “Understanding of the game” and a “Clear observation methodology” represented essential skills that tactical analysts need to put into practice. From these results, we emphasize the rigor and systematization that should characterize the observation phase in order to detect patterns of behavior of our own team and of our opponents, with the aim of intervening afterward through the training for the preparation of the match. In addition, the causes and/or solutions that the surveyed analysts attributed to some of the predictive models of the case under study were: adaptability of the “Style of play” itself to the “Opponent”; and “Pressure after loss.” Therefore, a style of play that is able to adapt to the opponent, contextual factors, and adequate pressure after losing the ball have been considered key aspects to optimize the performance of a team.

## Data Availability Statement

The raw data supporting the conclusions of this article will be made available by the authors, without undue reservation.

## Ethics Statement

The studies involving human participants were reviewed and approved by Ethics Committee of the Research Center of Sport Sciences at University Pablo de Olavide, based at Seville (Spain) and conformed to the recommendations of the Declaration of Helsinki. The patients/participants provided their written informed consent to participate in this study.

## Author Contributions

RA-M: conceptualization, formal analysis, and visualization. RA-M and FO-S: methodology, investigation, and writing—original draft preparation. JG-J and RA-M: software. ÁR-G and FO-S: validation, resources, writing—review and editing, and data curation. FO-S and JG-J: resources and writing—review and editing. JG-J: supervision. ÁR-G: project administration. All authors have read and agreed to the published version of the manuscript.

## Conflict of Interest

ÁR-G was employed by football club “Watford Football Club”. The remaining authors declare that the research was conducted in the absence of any commercial or financial relationships that could be construed as a potential conflict of interest.

## Publisher's Note

All claims expressed in this article are solely those of the authors and do not necessarily represent those of their affiliated organizations, or those of the publisher, the editors and the reviewers. Any product that may be evaluated in this article, or claim that may be made by its manufacturer, is not guaranteed or endorsed by the publisher.
