# Supplementary material for: Perceptions of Football Analysts Goal-Scoring Opportunity Predictions: A Qualitative Case Study
Source: Front Psychol. 2021 Sep 6;12:735167. doi: 10.3389/fpsyg.2021.735167 (PMC8450592; doi:10.3389/fpsyg.2021.735167)
Supplement: Supplementary file 1 [file Data_Sheet_1.PDF]

INTERVIEW

Part 1: GENERAL

- How important do you consider match analysis (both your team's and your opponents') in achieving success with your team?

- What characteristics do you consider essential for a coach/analyst to provide effective analysis?

- What aspects do you attach most importance to when analysing opposing teams and why?

- How do you think he changes the way he analyses the match when a match is in progress to the way he analyses it afterwards?

- Nowadays through. Mediacoach, Instat, Wyscout, Opta and other analysis platforms and data providers, we obtain data on collective and individual performance, both conditional and technical-tactical. Which are the most interesting variables or indicators to take into account? Which ones do you consider less relevant? Which ones do you think should be added? Do you use these performance indicators when designing training tasks? In what way?

- There is a trend towards data analysis, but averages, percentages and very basic indicators are still being used. What is the next step to bring value to training and competition?

Part 2: SPECIFIC

- Model 1:
  - Why do you think this probability is higher at the beginning of both halves than at the end of the match?

  - What pre-game strategy would you use to reduce the likelihood of conceding a goal-scoring chance when the ball is lost in your own half at the start of the 1st and 2nd half?

  - During the match would the solution or intervention be different if you detect such a weakness?

- Model 2:
  - Why do you think that a low duration and low number of passes is so decisive for the opponent in order to generate scoring chances?

  - What strategy would you use beforehand to avoid conceding a goal-scoring chance after losing the ball in your own half?
    would be to occupy the field rationally in order to be able to "defend with the ball".

  - What modifications or indications would you give if you observe that your team is having numerous losses in their own half?

- Model 3:
  - If the statistics speak of a greater probability of scoring goals with short attacks and few passes, why is so much importance given to possession of the ball and why are there continuous tasks to maintain possession?
  - Why do you think there is more stealing in the 2nd half?
  - Why do you think that the opponent's theft generates more danger than the interception of a pass?
  - What pre-game strategy would you use to avoid conceding a goal-scoring chance after a steal, especially in the 2nd half?
  - What modifications or indications would you give if you observe that your team is experiencing numerous theft losses?

- Model 4:
  - Why do you think the momentary result of the match has an influence on whether the chance is converted into a goal?
  - What pre-emptive strategy would you use to avoid conceding a goal-scoring chance if your team were to take the lead?
  - What modifications or indications would you give if the team takes the lead?
